# Prescription of Kampo Formulations for Pre-natal and Post-partum Women in Japan: Data From an Administrative Health Database

**DOI:** 10.3389/fnut.2021.762895

**Published:** 2021-11-12

**Authors:** Satoko Suzuki, Taku Obara, Tomofumi Ishikawa, Aoi Noda, Fumiko Matsuzaki, Ryutaro Arita, Minoru Ohsawa, Nariyasu Mano, Akiko Kikuchi, Shin Takayama, Tadashi Ishii

**Affiliations:** ^1^Department of Education and Support for Regional Medicine, Tohoku University Hospital, Sendai, Japan; ^2^Department of Kampo Medicine, Tohoku University Hospital, Sendai, Japan; ^3^Department of Pharmaceutical Sciences, Tohoku University Hospital, Sendai, Japan; ^4^Division of Molecular Epidemiology, Department of Preventive Medicine and Epidemiology, Tohoku Medical Megabank Organization, Tohoku University, Sendai, Japan; ^5^Laboratory of Clinical Pharmacy, Tohoku University Graduate School of Pharmaceutical Sciences, Sendai, Japan; ^6^Department of Obstetrics and Gynecology, Tohoku University Hospital, Sendai, Japan

**Keywords:** herbal medicine, Japanese traditional medicine, Kampo medicine, pregnant women, database

## Abstract

**Introduction:** Traditional Japanese (Kampo) medicines are often prescribed for women in Japan before, during, and after pregnancy. However, detailed information on the actual frequency of use and safety of Kampo preparations during pregnancy is lacking.

**Aim:** To investigate the frequency of prescription of Kampo medicines for pregnant women in Japan.

**Methods:** Information on Kampo medicines prescribed during outpatient care and hospitalization of selected mothers from January 2005 to August 2016 were extracted from the Japan Medical Data Center (Tokyo, Japan), which is a large claims database.

**Results:** Of the 33,941 subscribers identified from the database, 16,294 (48%) received at least one prescription of a Kampo medicine. Kakkonto was the formula most prescribed during the study period, followed by shoseiryuto and tokishakuyakusan. In the 180 days before pregnancy, the most prescribed medicine was tokishakuyakusan, followed by kakkonto and shoseiryuto. Shoseiryuto, tokishakuyakusan, and kakkonnto were the formulae most prescribed during pregnancy. The most prescribed medicines during the 180 days postpartum were kakkonto, shoseiryuto, and saireito.

**Conclusions:** Information in the Japanese insurance system shows that Kampo medicines are often prescribed during pregnancy. Most of these prescriptions are generally used for the treatment of common cold. Tokishakuyakusan in particular is usually used in the treatment of various symptoms of pregnancy. Further research is needed to clarify the relationship between the use of Kampo medicines during pregnancy and adverse events in infants in Japan.

## Introduction

Traditional Japanese (Kampo) medicines are used in combination with various herbal plants that have complementary medicinal properties. In Japan, the current health insurance system covers prescription of Kampo medicines, available as both herbs for decoctions and extract formulations. Kampo formulations are commonly prescribed by medical doctors under the Japanese health insurance system, both during hospitalization and in outpatient settings (1–3). The most prescribed Kampo formulae in the outpatient setting include kakkonto, shoseiryuto, and maoto (4, 5). In the study of prescriptions, including inpatient and outpatient, shakuyakukanzoto, kakkonto, and daikenchuto were the most commonly prescribed (6). Many Kampo medicines have been recommended for the treatment of symptoms or disorders in several clinical practice guidelines in Japan (7, 8). However, the women mentioned in these guidelines are not pregnant women. Kampo medicines, such as tokishakuyakusan, kamishoyosan, and keishibukuryogan, are commonly prescribed for Japanese women (4). Tokishakuyakusan in particular has been shown to be effective for dysmenorrhea (9, 10). In addition, Kampo medicines, including tokishakuyakusan and saireito, may improve live birth rate among women with recurrent pregnancy loss (11). Kampo medicines, including shohangekabukuryoto, toukishakuyakusan, and hangekobokuto, are prescribed for pregnant women with hyperemesis gravidarum, and may reduce unplanned admissions and medical costs for them (12). Furthermore, it has been shown that tokishakuyakusan, saireito, and boiogito improve hypertension and intrauterine growth restriction in preeclampsia rats (13, 14). Kyukichoketsuin, another Kampo medicine, is prescribed to stabilize the psychological state during the postpartum period (15). Kyukichoketsuin is also presumed to improve lactation during the postpartum period (16).

The drug information for most Kampo medicines states that the safety of the medicine in pregnancy has not been established (17). Furthermore, the drug information also indicate that Kampo medicines containing rhubarb [*Rheum palmatum* Linné, *Rheum tanguticum* Maximowicz, *Rheum officinale* Baillon, *Rheum coreanum* Nakai (*Polygonaceae*); rhizome] (18), peach kernel [*Prunus persica* Batsch, *Prunus persica* Batsch var, *davidiana* Maximowicz (*Rosaceae*); seed] (19), moutan bark [*Paeonia suffruticosa* Andrews (*Paeoniaceae)*; root] (20), safflower [*Carthamus tinctorius* Linné (*Compositae*); flower], achyranthes root [*Achyranthes bidentata* Blume. *Achyranthes fauriei* H. Léveillé et Vaniot (*Amaranthaceae*); root] (20), and anhydrous sodium sulfate (19, 21) may cause premature miscarriage and should not be administered to pregnant women or women who may be pregnant. In addition, processed aconite root (*Aconitum carmichaeli* Debeaux. *Aconitum japonicum* Thunberg (*Ranunculaceae*) and tuberous root) (20) are more likely to have side effects (premature birth or abortion) in pregnant women. Rhubarb may also cause diarrhea in infants; therefore, it should be administered with caution to women who are breastfeeding (18).

To the best of our knowledge, there is currently no literature on the adverse effects of herbal medicines during pregnancy in humans. In addition, information on the actual frequency of use and safety of Kampo preparations during pregnancy in Japan is lacking. Therefore, we investigated the frequency of the prescription of Kampo medicines during pregnancy, as well as during the prenatal and postpartum periods, in the current Japanese insurance system using a large health insurance claims database.

## Materials and Methods

### Data Source

The data used for this study were extracted from the Japan Medical Data Center Claims Database (JMDC, Tokyo, Japan) (22). The JMDC is a database of health insurance claims and health examination results in Japan. The database stores anonymous data provided by employer health insurance groups. Subscriber information includes sex, year and month of birth, and the period during which the data were obtained. All monthly medical claims data of outpatient, inpatient, and pharmacy services covered by health insurance are recorded in the database. This includes diagnoses, consultations, drugs, and procedures. Diagnoses are recorded based on the International Classification of Diseases 10th Revision codes and the Japanese standardized diagnosis codes. Information on whether the diagnosis was considered the main diagnosis or whether the diagnosis was suspected or confirmed is also recorded. Drugs are classified according to the Anatomical Therapeutic Chemical Classification System and the Japanese code for reimbursement.

This study was approved by the institutional review board of Tohoku University School of Medicine on July 19, 2016 (registration number: 2016-1-230). Since the data were de-identified, the requirement for informed consent was waived.

### Participants

**Figure 1:**
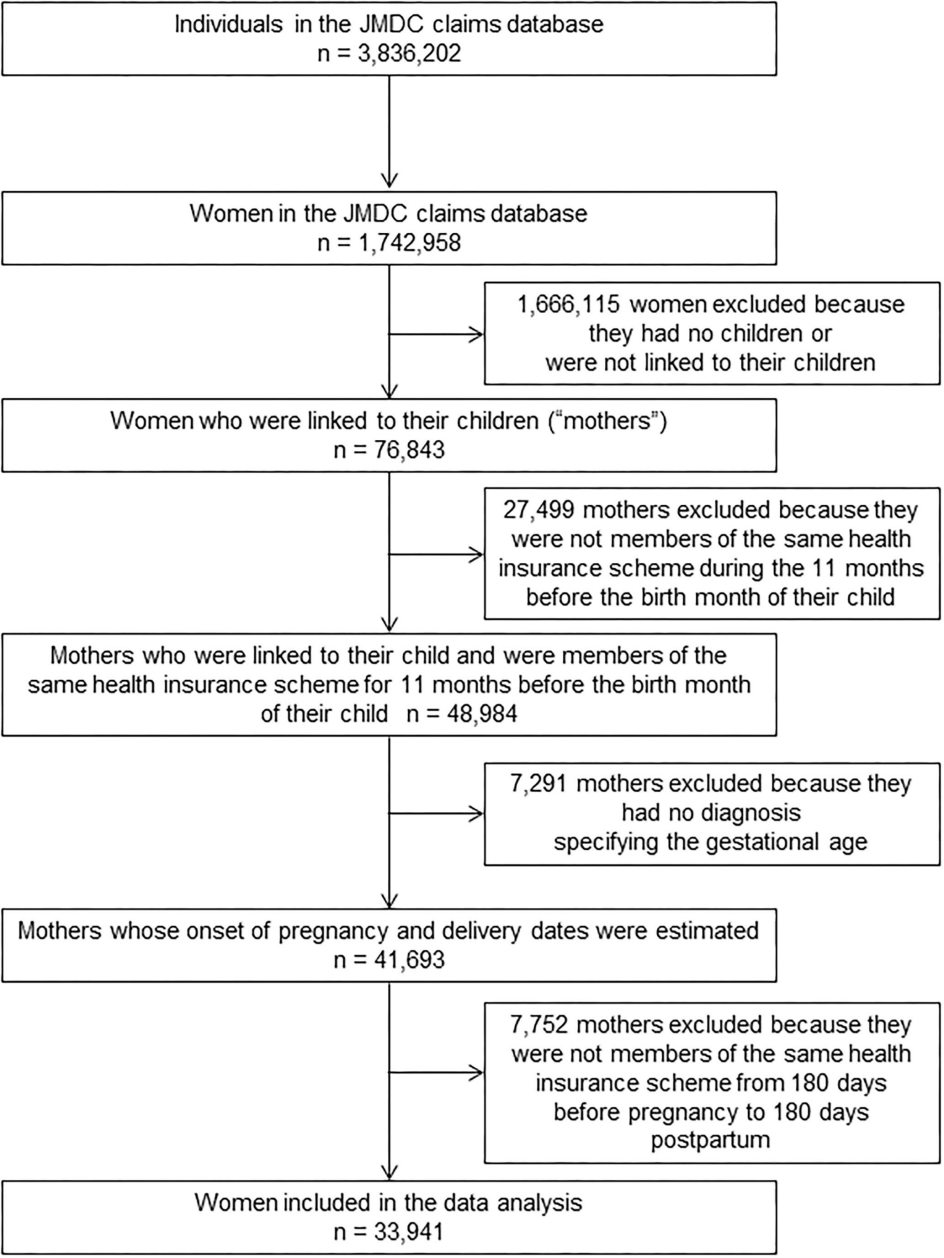
Flowchart of patient selection for inclusion in this study.

The dataset that was available on February 27, 2017, was used for this study. This dataset included 3,836,202 men and women covered by health insurance between January 2005 and August 2016. Mothers whose children were born and could be identified were included. Mothers can be linked with their offspring in the JMDC claims database if their infants are enrolled with the same health insurer. This allows the identification of the month and year of birth (the date of birth is not available to avoid re-identification). Women who met the following eligibility criteria were selected: (1) women who can be linked to a child whose birth month and month of enrollment in the health insurance scheme are the same; (2) women who were enrolled in the same health insurance company during the 11 months prior to the month of the child's birth; (3) women whose dates of pregnancy onset and delivery could be estimated; and (4) women who were members of an health insurance scheme exclusively from 180 days before the onset of pregnancy to 180 days postpartum. The flowchart of the patient selection process is shown in Figure 1. Prescription records were summarized at the individual level to calculate the frequency of prescription.

### Estimation of Dates of Pregnancy Onset and Delivery

Dates of pregnancy onset or delivery are not available in the Japanese claims database. Thus, the dates were estimated using algorithms described elsewhere (23–25).

The date of pregnancy onset was estimated by subtracting the gestational age recorded as part of the diagnosis at a specific visit from the date of the diagnosis. For example, if “delivery at week 39” was recorded, 39 weeks and 0 days were subtracted. If a woman made several visits with diagnoses specifying her gestational age, the longest gestational age was utilized. This is because the gestational age at delivery represents the best obstetric estimate for clinical care (25–27); thus, the gestational age recorded in the later stages of pregnancy is more accurate. An assessment conducted using administrative data from a university hospital in Japan showed that 92.8% of the estimated dates of pregnancy onset were within ±7 days of the gold standard date of pregnancy onset (28).

The date of delivery was estimated based on delivery-related entries described in our previous reports and the birth months of the infants (23–25). We utilized an algorithm in which the earliest dates of the selected diagnoses and surgical procedures were regarded as delivery dates (23–25). In cases without such diagnoses or procedures, the earliest dates of any other delivery-related entries, including diagnoses, surgical or medical procedures, or injectable medications, were considered the delivery dates. An assessment conducted using administrative data from a university hospital in Japan indicated that 96.4% of the estimated delivery dates were within ±7 days of the gold standard delivery date (28). Given that information on birth months and years are available, delivery-related entries were used only when the date was within the infant's birth month. Delivery is not always covered by health insurance in Japan, and related information is not recorded when a delivery does not require procedures, medications, etc., that are covered by health insurance. In such cases, the 15th day of the neonatal month was considered the delivery date.

A pregnancy that reaches or extends beyond 294 days of gestation is considered a post-term pregnancy, and labor induction is recommended due to an increased risk of perinatal mortality (27). Thus, if the difference between the estimated dates of pregnancy onset and delivery exceeded 294 days, a gestational period of 294 days was uniformly assigned and onset of pregnancy was considered to be 294 days before the estimated delivery date.

### Analysis of Prescriptions

Data on the Kampo medicines prescribed to the sampled mothers during outpatient care and hospitalization were extracted based on the Anatomical Therapeutic Chemical Classification System code V03AX. The dispensing date was used to estimate the duration of exposure to the prescribed drugs; if the data was unavailable, the hospitalization date was used instead. As the month and year were available for each claim, the 15th day of the month was assigned to a drug if neither the dispensing date nor the hospitalization date was available. Information on the days of supply for each prescription were available and were used to estimate the duration of exposure.

The prescription frequency for Kampo medicines was determined for the 180 days before pregnancy, during pregnancy, and the 180 days postpartum. A Kampo medicine was counted as one if the medicine was prescribed at least once before pregnancy, during pregnancy, or during the postpartum period, to avoid duplication of long-term administration. If two or more Kampo medicines were prescribed at the same time, each medicine was counted as one. Crude Kampo drugs used for decoctions, as well as extracts, were included in this study. All data were analyzed using SAS version 9.4 (SAS Institute, Inc., Cary, NC, USA).

## Results

Of the 3,836,202 men and women covered by health insurance between January 2005 and August 2016, 33,941 women who met our eligibility criteria were identified and included in the analysis (Figure 1). Delivery dates were estimated based on the earliest dates of selected diagnoses and surgical procedures for 12,604 women, the earliest dates of any other delivery-related entries for 5,014 women, and the 15th day of the neonatal month for 16,323 women. A period of 294 days was uniformly assigned as the gestational period for 569 women (1.7%) whose gestational period exceeded this duration. There were 119,521 records of Kampo medicines prescribed for the 33,941 women. Duration of exposure was evaluated from 103,285 (86.4%) and 1,614 (1.4%) dispensing and hospitalization dates, respectively. For the remaining 14,622 records (12.2%), the 15th day of the month and the year recorded in each claim were used. The mean age at delivery and the duration of gestation were 32.3 years [standard deviation (SD): 4.5 years] and 270.1 days (SD: 13.5 days), respectively. A total of 16,294 subscribers (48%) received at least one prescription of Kampo extract formulations.

The Kampo formulae included in the analysis are those with a prescription frequency of 0.4% or more (Table 1). Kakkonto was the formula prescribed to the largest number of patients during the study period (5,075 patients, 15.0%), followed by shoseiryuto (4,833, 14.2%), tokishakuyakusan (3,162, 9.3%), bakumondoto (2,856, 8.4%), and saireito (1,522, 4.5%). In the 180 days before pregnancy, the medicine most frequently prescribed was tokishakuyakusan (781, 2.3%), followed by kakkonto (678, 2.0%), shoseiryuto (526, 1.5%), bakumondoto (412, 1.2%), and unkeito (244, 0.7%). The medicine most frequently prescribed during pregnancy was shoseiryuto (3,629, 10.7%), followed by tokishakuyakusan (2,724, 8.0%), kakkonnto (2,535, 7.5%), bakumondoto (2,043, 6.0%), and saireito (1,000, 2.9%). The most prescribed medicine during the 180 days postpartum was kakkonto (2,605, 7.7%), followed by shoseiryuto (1,379, 4.1%), saireito (873, 2.6%), bakumondoto (741, 2.2%), and goreisan (422, 1.2%). The prescription status of crude drugs used in decoctions is shown in Table 2; only those with a prescription frequency of 0.1% or more are listed.

**Table 1:** Kampo formulae with a prescription frequency of 0.4% or more.

**Kampo formula**	**Total**		**Pre-pregnancy**		**Pregnancy**		**Post-partum**	
	***n***	**(%)**	***n***	**(%)**	***n***	**(%)**	***n***	**(%)**
Kakkonto	5,075	(15.0)	678	(2.0)	2,535	(7.5)	2,605	(7.7)
Shoseiryuto	4,833	(14.2)	526	(1.5)	3,629	(10.7)	1,379	(4.1)
Tokishakuyakusan	3,162	(9.3)	781	(2.3)	2,724	(8.0)	311	(0.9)
Bakumondoto	2,856	(8.4)	412	(1.2)	2,043	(6.0)	741	(2.2)
Saireito	1,522	(4.5)	125	(0.4)	1,000	(2.9)	873	(2.6)
Goreisan	994	(2.9)	228	(0.7)	500	(1.5)	422	(1.2)
Shohangekabukuryoto	612	(1.8)	14	(0.0)	597	(1.8)	8	(0.0)
Maoto	567	(1.7)	187	(0.6)	182	(0.5)	224	(0.7)
Maobushisaishinto	551	(1.6)	207	(0.6)	162	(0.5)	255	(0.8)
Kikyoto	539	(1.6)	129	(0.4)	239	(0.7)	239	(0.7)
Kakkontokasenkyusin'i	528	(1.6)	155	(0.5)	245	(0.7)	196	(0.6)
Hangekobokuto	480	(1.4)	82	(0.2)	366	(1.1)	74	(0.2)
Daikenchuto	412	(1.2)	47	(0.1)	154	(0.5)	300	(0.9)
Shakuyakukanzoto	374	(1.1)	88	(0.3)	249	(0.7)	78	(0.2)
Rikkunshito	357	(1.1)	96	(0.3)	213	(0.6)	89	(0.3)
Hochuekkito	280	(0.8)	110	(0.3)	86	(0.3)	130	(0.4)
Unkeito	271	(0.8)	244	(0.7)	129	(0.4)	4	(0.0)
Shosaikotokakikyosekko	263	(0.8)	83	(0.2)	113	(0.3)	103	(0.3)
Keishibukuryogan	234	(0.7)	129	(0.4)	85	(0.3)	80	(0.2)
Keishito	224	(0.7)	46	(0.1)	118	(0.3)	72	(0.2)
Kamishoyosan	219	(0.6)	155	(0.5)	78	(0.2)	53	(0.2)
Kyukichoketsuin	213	(0.6)	22	(0.1)	70	(0.2)	181	(0.5)
Goshuyuto	201	(0.6)	36	(0.1)	151	(0.4)	39	(0.1)
Hangeshashinto	190	(0.6)	53	(0.2)	97	(0.3)	48	(0.1)
Saikokeishito	186	(0.5)	66	(0.2)	71	(0.2)	69	(0.2)
Mashiningan	186	(0.5)	17	(0.1)	147	(0.4)	59	(0.2)
Choreito	173	(0.5)	53	(0.2)	60	(0.2)	66	(0.2)
Shishihakuhito	163	(0.5)	46	(0.1)	76	(0.2)	63	(0.2)
Ninjin'yoeito	158	(0.5)	13	(0.0)	118	(0.3)	70	(0.2)
Kososan	155	(0.5)	25	(0.1)	94	(0.3)	45	(0.1)
Makyokansekito	148	(0.4)	33	(0.1)	79	(0.2)	54	(0.2)
Otsujito	142	(0.4)	14	(0.0)	75	(0.2)	91	(0.3)
Kikyosekko	138	(0.4)	56	(0.2)	50	(0.1)	58	(0.2)

**Table 2:** The prescription status of crude drugs used in decoctions (those with a prescription frequency of 0.1% or more).

**Crude drug for Kampo decoction**		**Total**		**Pre-pregnancy**		**Pregnancy**		**Post-partum**	
**Plant name**	**Plant part**	***n***	**(%)**	***n***	**(%)**	***n***	**(%)**	***n***	**(%)**
*Poria cocos* Wolf (*Polyporaceae*)	–	61	(0.2)	28	(0.1)	42	(0.1)	16	(0.0)
*Glycyrrhiza uralensis* Fisch.*Glycyrrhiza glabra* Linne (*Legminosae*)	Root	58	(0.2)	29	(0.1)	37	(0.1)	20	(0.1)
*Paeonia lactiflora* Pall. (*Paeoniaceae*)	Root	54	(0.2)	34	(0.1)	39	(0.1)	9	(0.0)
*Cinnamomum cassia* J. Presl (*Lauraceae*)	Cortex	44	(0.1)	24	(0.1)	26	(0.1)	9	(0.0)
*Cnidium officinale* Makino (*Umbelliferae*)	Rhizome	43	(0.1)	28	(0.1)	29	(0.1)	8	(0.0)
*Alisma orientale* Juzepczuk (*Alismataceae*)	Tuber	43	(0.1)	20	(0.1)	34	(0.1)	9	(0.0)
*Angelica acutiloba* Kitagawa. *Angelica acutiloba* Kitagawa var. *sugiyamae*Hikino (*Umbelliferae*)	Root	43	(0.1)	29	(0.1)	34	(0.1)	6	(0.0)
*Aconitum carmichaeli* Debeaux. *Aconitum japonicum* Thunberg (*Ranunculaceae*)	Tuber	40	(0.1)	25	(0.1)	27	(0.1)	5	(0.0)
*Atractylodes ovata* DenCandolle (*Compositae*)	Rhizome	38	(0.1)	17	(0.1)	29	(0.1)	8	(0.0)
*Panax ginseng* C. A. Meyer (*Panax schinseng* Nees) (*Araliaceae*) (Red Ginseng)	Root	35	(0.1)	25	(0.1)	24	(0.1)	4	(0.0)
*Zingiber officinale* Roscoe (*Zingiberaceae)* (Ginger)	Rhizome	34	(0.1)	16	(0.0)	21	(0.1)	10	(0.0)
*Panax ginseng* C. A. Meyer(*Panax schinseng* Nees) (*Araliaceae*) (Ginseng)	Root	31	(0.1)	18	(0.1)	21	(0.1)	9	(0.0)
*Atractylodes lancea* De Candolle. *Atractylodes chinensis* Koidzumi (*Compositae*)	Rhizome	30	(0.1)	15	(0.0)	20	(0.1)	8	(0.0)
*Ziziphus jujuba* Miller var. *inermis* Rehder (*Rhamnaceae*)	Fruit	29	(0.1)	11	(0.0)	19	(0.1)	10	(0.0)
*Bupleurum falcatum* Linné (*Umbelliferae*)	Root	27	(0.1)	11	(0.0)	17	(0.1)	10	(0.0)
*Gentiana lutea* Linné (*Gentianaceae*)	Root/Rhizome	25	(0.1)	12	(0.0)	8	(0.0)	11	(0.0)
*Rheum palmatum* Linné, *Rheum tanguticum* Maximowicz, *Rheum officinale* Baillon, *Rheum coreanum* Nakai (*Polygonaceae*)	Rhizome	25	(0.1)	11	(0.0)	14	(0.0)	5	(0.0)
*Pinellia ternata* Breitenbach (*Araceae*)	Tuberous root	23	(0.1)	11	(0.0)	16	(0.0)	5	(0.0)
*Rehmannia glutinosa* Liboschitz var. *purpurea* Makino. *Rehmannia glutinosa* Liboschitz (*Scrophulariaceae*)	Root	22	(0.1)	16	(0.0)	14	(0.0)	4	(0.0)
*Crocus sativus* Linné (*Iridaceae*)	Stigma	21	(0.1)	16	(0.0)	11	(0.0)	3	(0.0)
*Zingiber officinale* Roscoe (*Zingiberaceae*) (Processed Ginger)	Rhizome	20	(0.1)	12	(0.0)	11	(0.0)	7	(0.0)
*Scutellaria baicalensis* Georgi (*Labiatae*)	Root	18	(0.1)	5	(0.0)	10	(0.0)	7	(0.0)

Kampo medicines containing crude drugs (rhubarb, peach kernel, moutan bark, safflower, achyranthes root, anhydrous sodium sulfate, and processed aconite root), which should be administered cautiously in pregnancy, were also analyzed in this study. Only drugs with a prescription frequency of 0.1% or more were included. Regarding Kampo medicines used as laxatives, mashiningan, which contains rhubarb, was prescribed to 147 (0.4%) women, daiokanzoto to 82 (0.2%) women, otsujito to 75 (0.2%) women, bofutsushosan to 53 (0.2%) women, and tokakujokito to 29 (0.1%) women, and junchoto to 18 (0.1%) women during pregnancy. For Kampo medicines that contain peach kernel, keishibukuryogan was prescribed to 85 (0.3%) women, kyukichoketsuin to 70 (0.2%) women, tokakujokito to 29 (0.1%) women, and junchoto to 18 (0.1%) women during pregnancy. For Kampo medicines that contain moutan bark, keishibukuryogan was prescribed to 85 (0.3%) women, unkeito to 129 (0.4%) women, kamishoyosan to 78 (0.2%) women, and kyukichoketsuin to 70 (0.2%) women during pregnancy. Regarding Kampo medicines that contain processed aconite root, maobushisaishinto was prescribed to 162 (0.5%) women during pregnancy. For Kampo medicines that contain safflower, kyukichoketsuin, was prescribed to 70 (0.2%) women during pregnancy. For Kampo medicines that anhydrous sodium sulfate, bofutsushosan was prescribed to 53 (0.2%) women and tokakujokito to 29 (0.1%) women during pregnancy.

## Discussion

The present study involved the investigation of the frequency of the prescription of Kampo extract formulations in Japan using a large-scale claims database. Of the 3.8 million subscribers in the database, 33,941 mothers were sampled and 48% of them were prescribed herbal medicines. It has been reported that herbal medicines are widely used worldwide to treat a variety of ailments during pregnancy (29–31). However, there has been no comprehensive and detailed study on the use of Kampo medicine or herbal medicine during pregnancy. Previous birth cohort studies conducted using the data of the Japan Environment and Children's Study showed that Kampo medicines are used to treat common colds during pregnancy (32). The present study highlighted the specific names of Kampo medicines prescribed during pregnancy and the frequency of their prescription for prenatal and postpartum women. The prescription patterns differed slightly according to the duration of the pregnancy. The results of the present study showed that kakkonto and shoseiryuto, which are used for the treatment of common cold, were prescribed more frequently in the prenatal and postpartum periods. This is similar to the results of a previous study of Japanese outpatients (4). In contrast, tokishakuyakusan was commonly prescribed during pregnancy. According to the drug information of tokishakuyakusan (33), the medicine is used to treat various symptoms during pregnancy. The detailed mechanism by which Kampo medicines treat ailments has not yet been elucidated. However, the efficacy of tokishakuyakusan for treatment of ailments during pregnancy has been reported in small case reports (34). In addition, the findings of a previous cohort study conducted using the JMDC claims database suggest that tokishakuyakusan and saireito may improve fertility rate in patients with recurrent pregnancy loss (11). In the present study, we found that tokishakuyakusan is used frequently in pregnancy.

Kampo medicines that should be administered with caution in pregnancy were also prescribed to the women included in the present study. Several Kampo medicines used as laxatives are prescribed during pregnancy as pregnant women are prone to constipation (35). As previously mentioned, herbal medicines containing rhubarb should not be administered during pregnancy or lactation. This is because the main component of rhubarb, emodin (1,3,8-trihydroxy-6-methylanthraquinone), can induce embryonic cytotoxicity and as such should be used with caution in pregnancy (36, 37).

To our knowledge, no previous studies have shown the specific side effects or efficacy of Kampo crude drugs other than rhubarb that should be administered with caution during pregnancy (peach kernel, moutan bark, safflower, achyranthes root, anhydrous sodium sulfate, and processed aconite root) (19–21). We were able to determine the actual prescription status of these drugs in the present study; however, we could not assess their safety.

This study has several limitations. First, we could not determine if the women actually used the Kampo medicines prescribed. This may lead to a potential underestimation of the risks. However, the use of prescription data has the advantage of eliminating recall bias associated with self-reported data. Second, data on Kampo medicines purchased outside health insurance could not be obtained. Some Kampo medicines may have been purchased as over-the-counter drugs. Third, side effects of prescribing Kampo medicines to pregnant women could not be analyzed in this study. Thus, the safety of the prescribed Kampo medicines was not shown. Third, the results of this study did not show whether the quality of life of the pregnant women actually improved as a result of being prescribed Kampo medicine before, during, and after pregnancy. Fourth, the study population included only women with live-born children who were enrolled with the same health insurer. Therefore, it was not possible to evaluate the Kampo medicines prescribed to women whose pregnancy ended in abortion or stillbirth.

## Conclusion

Information in the Japanese insurance system demonstrates that Kampo medicines are often prescribed during pregnancy. Most of these prescriptions are drugs used for the treatment of common cold. Tokishakuyakusan in particular is used for the treatment of various symptoms of pregnancy. Further research is needed to clarify the relationship between the use of Kampo medicine during pregnancy and adverse events in infants in Japan.

## Data Availability Statement

The original contributions presented in the study are included in the article/supplementary material, further inquiries can be directed to the corresponding author/s.

## Ethics Statement

The studies involving human participants were reviewed and approved by the Institutional Review Board of Tohoku University School of Medicine. Written informed consent for participation was not required for this study in accordance with the national legislation and the institutional requirements.

## Author Contributions

SS: methodology, formal analysis, and writing—original draft. TO: conceptualization, methodology, supervision, writing, review, and editing. TIshik, AN, and FM: methodology, formal analysis, investigation, data curation, review, and editing. MO and RA: methodology, formal analysis, review, and editing. ST: funding acquisition, review, and editing. NM, AK, and TIshii: review and editing. All authors contributed to the manuscript and approved the submitted version.

## Funding

This work was supported by grants from the Ministry of Health, Labour, and Welfare of Japan (H23-iyaku-ippan-006) and the Japan Society for the Promotion of Science (19K09746 and 20K16070).

## Conflict of Interest

ST, AK, MO, and TIshii belong to the Department of Kampo and Integrative Medicine at Tohoku University School of Medicine. The department received a grant from Tsumura & Co., a Japanese manufacturer of Kampo medicine; however, the grant was used according to the rules of Tohoku University. Potential conflicts of interests were addressed by the Tohoku University Benefit Reciprocity Committee and were managed appropriately. TIshik is an employee of Pfizer R&D Japan. TIshik is also a research collaborator at Tohoku University and contributed to the present study independently of Pfizer R&D Japan. The remaining authors declare that the research was conducted in the absence of any commercial or financial relationships that could be construed as a potential conflict of interest.

## Publisher's Note

All claims expressed in this article are solely those of the authors and do not necessarily represent those of their affiliated organizations, or those of the publisher, the editors and the reviewers. Any product that may be evaluated in this article, or claim that may be made by its manufacturer, is not guaranteed or endorsed by the publisher.
